# Precision Seeding Compensation and Positioning Based on Multisensors

**DOI:** 10.3390/s22197228

**Published:** 2022-09-23

**Authors:** Jiaze Sun, Yan Zhang, Yuting Zhang, Peize Li, Guifa Teng

**Affiliations:** 1 School of Information Science and Technology, Hebei Agricultural University, Baoding 071000, China; 2 Hebei Key Laboratory of Agricultural Big Data, Baoding 071000, China

**Keywords:** curve seeding compensation, seeding position, multisensors, smart farming

## Abstract

The current multi-row planter always leads to uneven seeding spacing between rows while seeding in curve paths, which causes uneven growth, a cost increase of production and management, and reduced yield. With the development of smart farming technology, a curve seeding compensation and precise positioning model is proposed in the paper to calculate the real-time speed and position of each seeding unit based on the information from multisensors, such as GNSS and IMU, and to predict the next seeding position to achieve uniform seeding on the curve and improve the unit yield of crops. MATLAB Simulink simulation experiments show that the seeding pass rate of the model is 99.97% when the positioning accuracy is ±0.01 m and the traction speed is 1 m/s, and the seeding pass rate of the five-row seeder is as high as 99.81% when the traction speed is 3 m/s, which verifies the effectiveness and practicality of the model.

## 1. Introduction

Corn is one of the most important food crops in China, which is also an important industrial raw material in addition to its direct consumption use [1]. The yield per unit area of corn in China is only 6316.70 kg/ha [2] far lower than that in developed agricultural countries such as the United States. At present, the mainstream corn precision seeder in the domestic market adopts the design of whole system driven by the ground wheel, in which the seeding units run simultaneously at the same speed in all rows [3]. When the seeder sows in a curve in the field, the difference is formed in the trajectory between the seeding units in inner rows and those in outer rows due to their different rotation radius. However, the seeder is driven by the ground wheel [4], and the seeding speed is always the same in all the rows, resulting in relatively dense inner row seeding and relatively sparse outer row seeding in curve areas. Furthermore, it causes the uneven seeding spacing, thus leading to the increase of the seeding volume of seeds, the increase of subsequent management cost of plants, and the decrease of the overall yield [5,6].

Horner and Eslinge tracked and analyzed the growth process of 24 rows of corn seeded by the corn seeder, and pointed out that in the curve area, the seeding density of the inner row was 124% of that of the middle row, and the seeding density of the outer row was only 81% of that of the middle row [7]. The average yield of the middle rows was 673.184 kg/mu, and the average yields of the inner rows and the outer rows were 635.553 kg/mu and 547.746 kg/mu, respectively. Therefore, the yield per mu of land decreased by 37.631 kg and 125.438 kg, respectively, in the inner and outer rows. In a study, Ag Leader pointed out that the speed difference between the inner/outer row and the center of the seeder caused uneven seeding in each row, and that the corn yield per mu decreased by 21.324 kg for every inch of curve seeding [8]. It is necessary to develop a set of curve seeding compensation and positioning algorithm that can control the seeding speed of each seeding unit in real time to solve the yield loss caused by uneven seeding.

Replacing the mechanical transmission system of the seeder with the electric drive system is the premise of curve seeding compensation and positioning algorithm. An electric drive control system for the on-site setting of the seed discharge drive was designed by Yang Shuo et al. [9] to solve the problem that the existing precise electric drive seed discharge cannot realize fast regulation control; thus, there is a need to realize the setting and control of the rotation speed of the seed discharge plate. It is necessary to research the use of electric drive instead of mechanical drive to realize uniform seeding. A precise corn seeding monitoring system based on CAN bus was designed by Yanxin Yin et al. [10], which could monitor the position, running speed, and seeding frequency of the seeder in real time. A high-speed precise corn seeding control system navigated by Beidou system was designed by Weiwei Wang et al. [11] to solve the problem of low seeding efficiency and poor uniformity of traditional seeders, which took pneumatic corn seeder as the research object and incorporated the double closed-loop fuzzy PID algorithm of Beidou autopilot as the control core. The studies described above improved the seeding quality considerably and achieved independent control of the planter. However, the working speed detected by these methods is the working speed of the seeder and not the individual speed of each unit. Therefore, these methods can only be used as independent control of seeding and not directly for turn compensation.

To obtain the working speed of each seeding unit, one method is to install a speed sensor on each seeding unit to measure the working speed of each seeding unit. Another method is to deduce the working speed of each seeding unit by calculating the difference in ground wheel rotation speed. In order to improve the accuracy of measurement while reducing the complexity of hardware, Xiantao He et al. [12] designed a GPS-based turn compensation system, which relied on the seeding prescription maps to pre-determine the seeding position and then adjusted the seeding rate in real time according to the position information received from GPS to finally achieve turn seeding compensation. However, on the one hand, only one GPS receiver cannot accurately determine the position and attitude information of the seeder and, on the other hand, the seeding operation cannot be carried out flexibly when the seeding prescription map is relied on. A precise seeding compensation and positioning algorithm based on multi-sensors is proposed in this study in order to improve the flexibility and precision of seeding in curve area. With the synergy of the GNSS position data-receiving device and multiple sensors, such as the IMU901 angular transducer, it is feasible to obtain the heading angle and position of the seeder in real time and to calculate the seeding position and traction speed of each seeding unit, based on which the seeding rate can be adjusted. This algorithm can not only effectively improve the uniformity and precision of seeding in curve area, but also effectively reduce the investment of equipment, economy, and human resources in the seeding process.

This paper was conducted to achieve the aim by: (1) developing a curve seeding compensation and precise positioning model to improve seeding quality; (2) calculating the speed and position of each seeding unit in real time based on the information from multiple sensors, such as GNSS and IMU, and predicting the next seeding position to achieve uniform seeding on the curve; (3) building a simulation model to analyze the factors affecting the performance of turn compensation in order to optimize the operating parameters of the control system.

## 2. Materials and Methods

**Figure 1:**
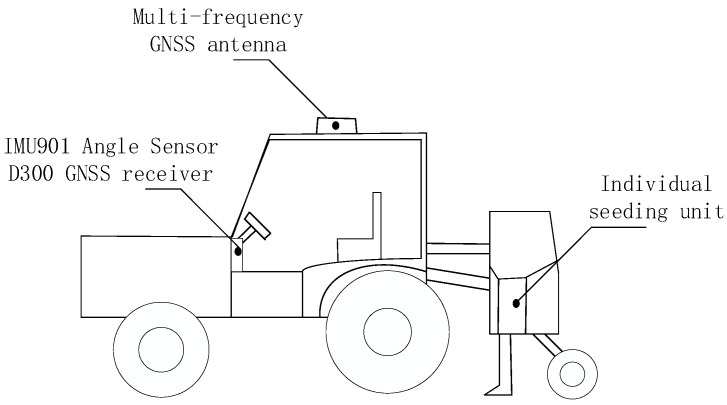
Overview of Device.

**Figure 2:**
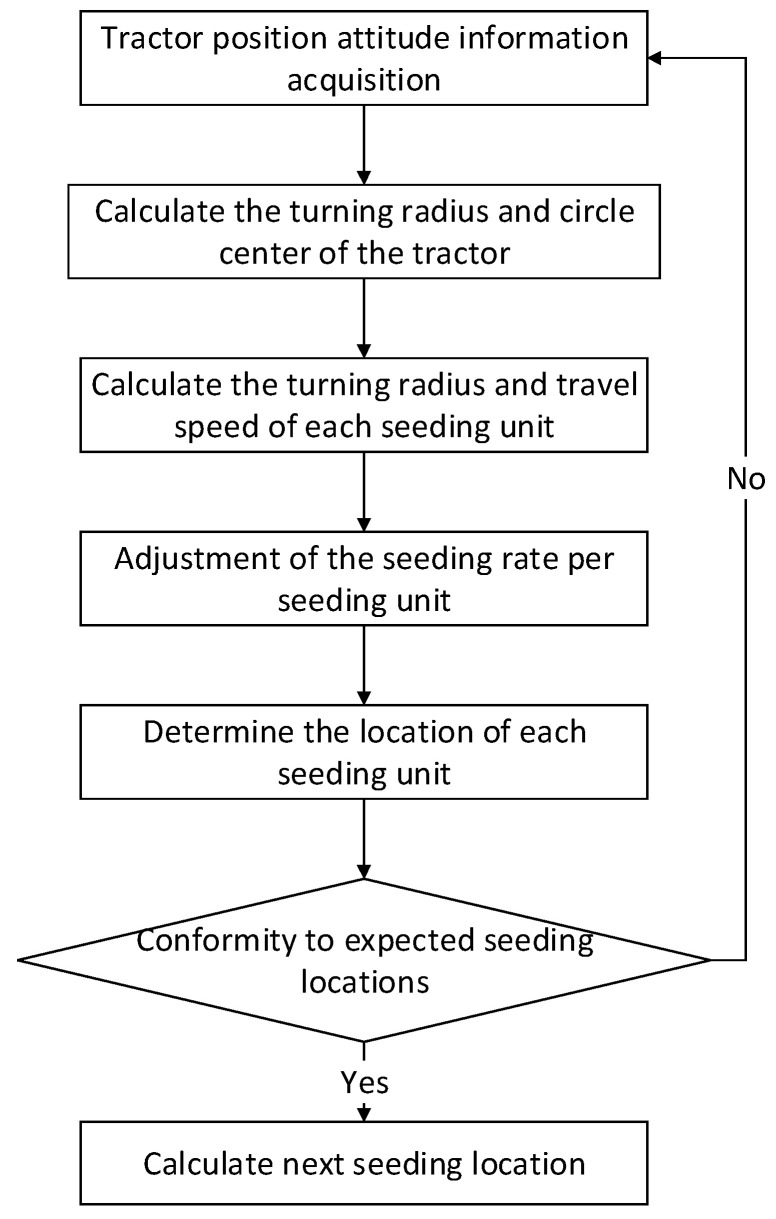
Algorithm Flow Chart.

As shown in Figure 1, the rigid connection was adopted between the tractor and the seeder in this study. When the tractor pulled the seeder to turn, the seeder and the tractor were regarded as the same rigid body system, that is, there was no relative deformation between the seeder and the tractor during the turning process. The real-time position data of the tractor could be acquired by the multi-satellite, multi-frequency GNSS antenna installed on the roof of the tractor and RTK auxiliary positioning device, but the measured position data were only the position data of the multi-satellite, multi-frequency GNSS antenna installed in the tractor [13], so it was necessary to calculate the position data of each seeding unit by taking into account of the rigid body motion principle [14]. With the position data and heading angle data received by the multi-satellite GNSS antenna and IMU901 angular transducer, it was feasible to calculate the turning radius and center position of tractor and to calculate the movement track of each seeding unit according to the position relationship between tractor and each seeding unit. The flow chart of the algorithm is shown in Figure 2.

### 2.1. Calculation of Turning Radius and Center of Tractor

**Figure 3:**
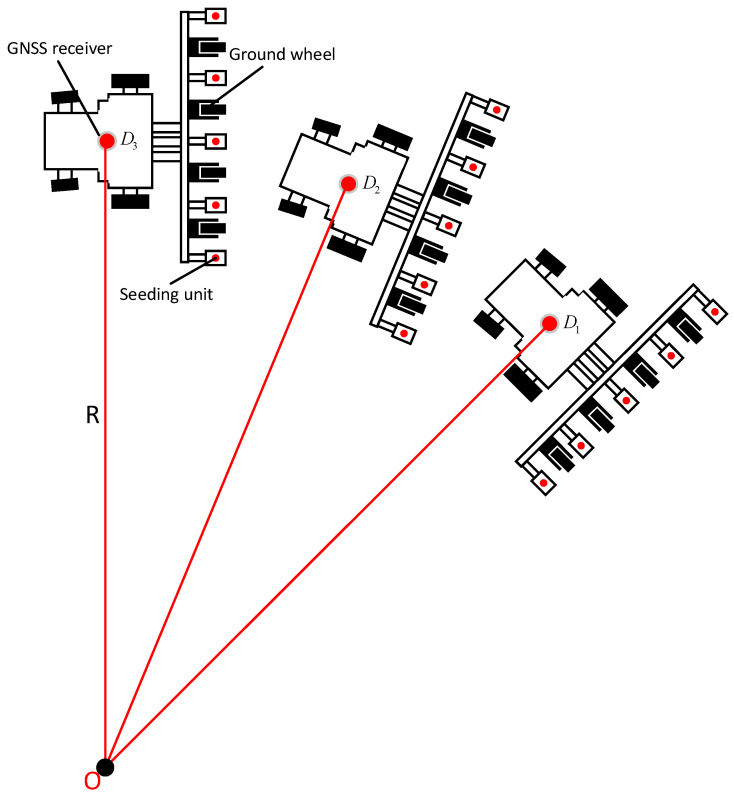
Tractor Steering Diagram.

The coordinate data received by the multi-satellite, multi-frequency GNSS antenna used in this study included longitude, latitude, elevation, and other three-dimensional coordinate data, but in agricultural production, the focus of calculation was mainly put on the longitude and latitude. Therefore, it was necessary to convert the received three-dimensional coordinate data into the coordinate data in plane coordinate system according to Mercator projection for further calculation [15]. The algorithm flow was as follows: Three latitude and longitude coordinates were continuously obtained; three consecutive plane coordinates were obtained by Mercator projection, i.e., $D_{1}\left(x_{1},y_{1}\right)$, $D_{2}\left(x_{2},y_{2}\right),$ and $D_{3}\left(x_{3},y_{3}\right)$, [16], as shown in Figure 3; and then the turning radius R and center position O of the moving track formed by the tractor position data receiving point were calculated with Formula (1) as below:

(1) $$R=\sqrt{{\left(x_{0}-x_{1}\right)}^{2}+{\left(y_{0}-y_{1}\right)}^{2}}$$

where $\left(x_{0},y_{0}\right)$ is the center coordinate, and R is the rotation radius (m) of the moving track formed by the receiver at the top installation position of the tractor.

Let $a=2\left(x_{2}-x_{1}\right),\;b=2\left(y_{2}-y_{1}\right),\;c=\left(x_{2}^{2}+y_{2}^{2}\right)-\left(x_{1}^{2}+y_{1}^{2}\right),\;d=2\left(x_{3}-x_{2}\right),\;e=2\left(y_{3}-y_{2}\right),\;f=\left(x_{3}^{2}+y_{3}^{2}\right)-\left(x_{2}^{2}+y_{2}^{2}\right)$; then, the coordinates of the center of the circle $\left(x_{0},y_{0}\right)$ can be deduced from Formula (2).

(2) $$\begin{array}{c} x_{0}=\frac{bf-ec}{bd-ea}\\ y_{0}=\frac{dc-af}{bd-ea} \end{array}$$

### 2.2. Calculation of Turning Radius of Individual Seeding Unit

**Figure 4:**
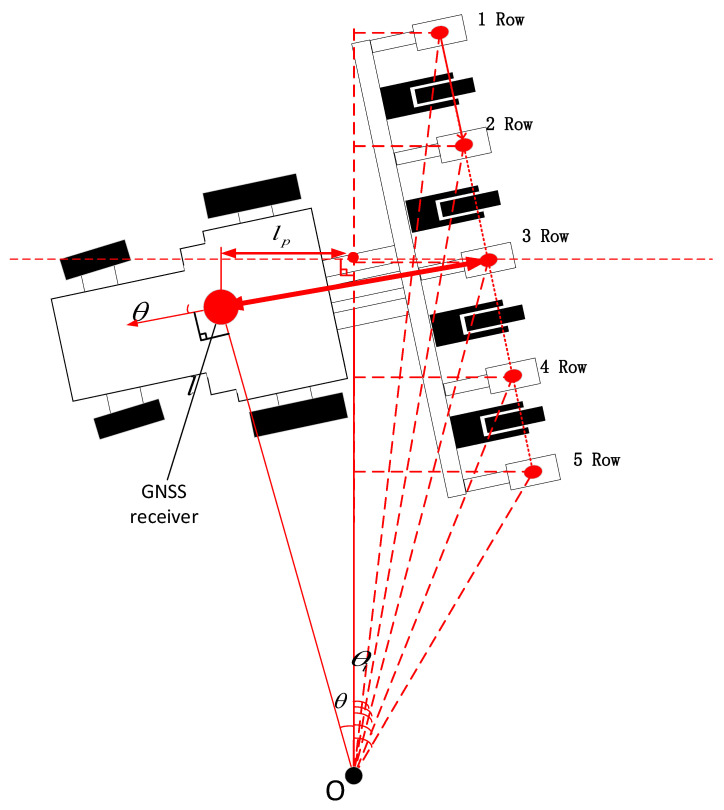
Calculation of Turning Radius.

As shown in Figure 4, when calculating the seeding radius of individual seeding unit, we should give priority to calculating the coordinates of the midpoint of the straight line formed by the seeding units, that is, the radius of seeding unit No.3 ($R_{3}\;$). The calculation was based on Formula (3):

(3) $$R_{3}=\sqrt{R^{2}+l^{2}}$$

where R is the turning radius of the receiver movement track, and l is the distance from the receiving point to the seeding unit No.3.

The calculation of the turning radius of individual seeding unit need to be based on $R_{3}$ and the heading angle data. It was the premise for calculation of $R_{i}$ to calculate the horizontal and vertical components $\left(R_{3x},R_{3y}\right)$ of $R_{3}$, with Formula (4) as below:

(4) $$\{\begin{array}{c} R_{3x}=\left(l-R\tan\theta \right)\cos\theta \\ R_{3y}=R/\sin\theta +\left(l-R\tan\theta \right)\sin\theta \end{array}$$

where θ is the heading angle.

The calculation of the rotation radius $R_{i}$ of individual seeding unit was calculated with Formulas (5) and (6):

(5) $$l_{i,n}=\left(\frac{n}{2}+1-i\right)l_{r}$$

(6) $$R_{i}=\sqrt{{\left(R_{3x}-l_{i,n}\sin\theta \right)}^{2}+{\left(R_{3y}+l_{i,n}\cos\theta \right)}^{2}}$$

where $l_{r}$ is the distance between every two seeding units, and $l_{i,n}$ is the distance between the i−th seeding unit and the middle seeding unit i∈[1,n].

According to the law of rotation of rigid body, it could be known that the individual seeding unit and tractor had the same angular velocity, so the traction speed of the seeder and the traction speed of individual seeding unit were calculated with Formulas (7) and (8), respectively, as below:

(7) $$V=\left(a/t_{0}+b/t_{0}+c/2t_{0}\right)/3$$

(8) $$v_{i}=\frac{R_{i}}{R}V\;i\in \left[1,n\right]$$

where V is the traction speed of tractor, and $v_{i}$ is the traction speed of individual seeding unit. The angular velocity $\omega_{i}$ of individual seeding unit was calculated with Formula (9) as below:

(9) $$\omega_{i}=\frac{v_{i}}{R_{i}}\;i\in \left[1,n\right]$$

### 2.3. Seeding Frequency of Individual Seeding Unit

**Figure 5:**
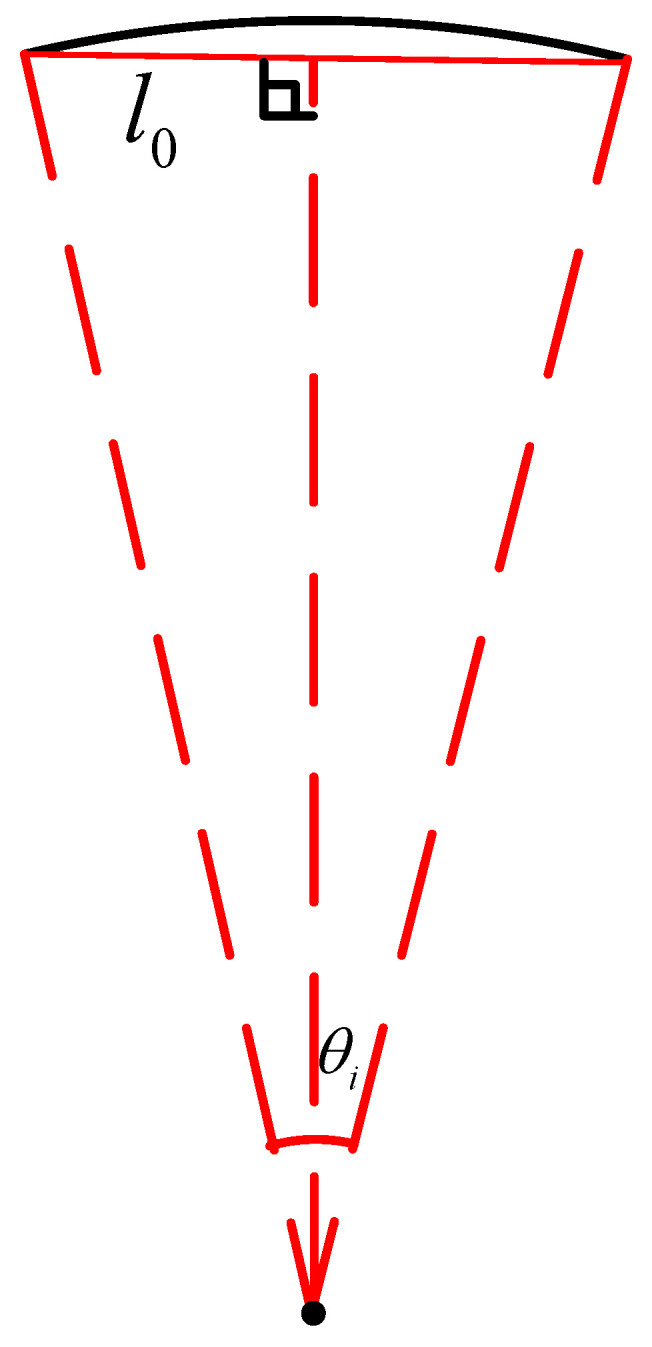
Calculation of Turning Angle.

When the seeder turned, the traction speed of the inner seeding unit was lower than that of the outer seeding unit. Therefore, it was necessary to adjust the seeding frequency of the individual seeding unit according to the different routes of the seeding units, in order to ensure the same seeding distance between plants. The seeding frequency required by each driving motor was determined by the specified seeding spacing $l_{0}$ and the traveling route of the corresponding seeding unit. As shown in Figure 5, it was the premise for calculation of seeding frequency to calculate the angle $\theta_{i}$ when the *i*-th seeding unit met the requirement about the seeding space l0, as shown in Formula (10):

(10) $$\theta_{i}=2\arcsin\left(\frac{l_{0}}{2R_{i}}\right)\;i\in \left[1,n\right]$$

So, the seeding frequency of the *i*-th seeding unit was calculated with Formula (11) as below:

(11) $$f_{i}=\frac{\omega_{i}}{\theta_{i}}\;i\in \left[1,n\right]$$

### 2.4. Positioning of Individual Seeding Unit

**Figure 6:**
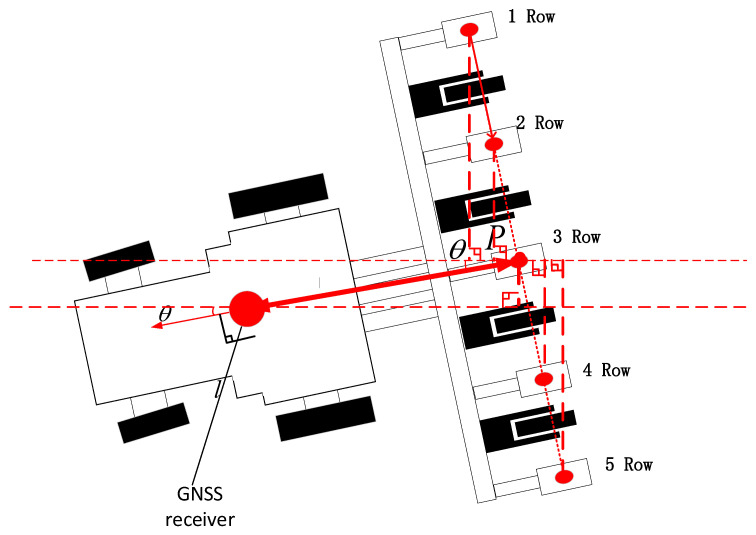
Calculation of Seeding Unit Position.

The falling position of the seeds of individual seeding unit shall be obtained in real time during the seeding process and recorded to the cloud server, in order to carry out the later management of corn more accurately. The schematic diagram of the algorithm for calculating the position of individual seeding unit in the seeding process is shown in Figure 6. The position between the position information receiver and the seeding unit were kept unchanged, so the positioning coordinate (x,y) of the middle seeding unit (point P) was calculated according to the received positioning coordinate (x,y) and heading angle (θ). According to the steering principle of rigid body, the heading angle of point P was θ. The position coordinate $\left(x_{p},y_{p}\right)$ of point P was calculated with Formula (12) as below:

(12) $$\{\begin{array}{c} x_{p}=x+l\cos\theta \\ y_{p}=y+l\sin\theta \end{array}$$

where the point P is the seed falling position of the middle seeding unit of the seeder, and l is the distance between the position of the receiver and the point P.

According to the position of the *i*-th seeding unit relative to point *p*, it was feasible to obtain the coordinate information $\left(x_{i},y_{i}\right)$ of the *i*-th seeding unit with Formula (13) as below:

(13) $$\{\begin{array}{c} x_{i}=x_{p}-l_{i,n}\sin\theta \\ y_{i}=y_{p}+l_{i,n}\cos\theta \end{array}\;i\in \left[1,n\right]$$

According to the coordinate information of the *i*-th seeding unit $\left(x_{i},y_{i}\right)$ and the position of the center of the circle $\left(x_{0},y_{0}\right)$, it was feasible to obtain the heading angle information ($\theta_{i}^{\prime }$) of the *i*-th seeding unit using Formula (14) as follows.

(14) $$\begin{array}{c} l=x_{i}-x_{0}\\ h=y_{i}-y_{0}\\ \theta_{i}^{\prime }=\arctan\frac{h}{l} \end{array}$$

According to the heading angle $\theta_{i}^{\prime }$ of the ith seeding unit and the rotation angle $\theta_{i}$ of the ith seeding unit in Formula (10) that conforms to the seeding spacing, it was possible to calculate the seeding angle $\theta_{i}^{\prime \prime }$ corresponding to the next seeding position, and the calculation Formula (15) is shown as follows.

(15) $$\theta_{i}^{\prime \prime }=\theta_{i}^{\prime }+\theta_{i}$$

The next seeding position $\left({x^{\prime }}_{i},{y^{\prime }}_{i}\right)$ of the *i*-th seeding unit was calculated with Formula (16) as below:

(16) $$\{\begin{array}{c} x_{i}^{\prime }=x_{0}-R_{i}\sin{\theta_{i}}^{\prime }\\ y_{i}^{\prime }=y_{0}+R_{i}\cos{\theta_{i}}^{\prime } \end{array}$$

where $\left(x_{0},y_{0}\right)$ is the center coordinate and $\left(x_{i},y_{i}\right)$ is the position of the *i*-th seeding unit i∈[1,n].

## 3. Results

### 3.1. Simulation Model

The curve seeding compensation and precise positioning model used in this study could calculate the real-time speed and position of individual seeding unit in each row according to the position and attitude data received by GNSS receiver and multiple sensors, and predict the next seeding position, so as to achieve the goal of uniform seeding in the curve area and improve the unit yield of crops. This simulation experiment was carried out with Simulink module in MATLAB (MathWorks Inc., Natick, Massachusetts, USA). Firstly, the simulation model of curve seeding was established in Simulink, and three variable factors were set, including traction speed, number of seeding rows, and positioning accuracy. Then, the analysis was conducted to determine the influence of three variable factors on the algorithm studied in this paper, and the comprehensive analysis was done on the seeding spacing after the application of this algorithm.

The simulation mainly consisted of the latitude and longitude information generation module, the position calculation module for the seeding unit in the center of the seeder and the position calculation module for individual seeding units. Among them, the latitude and longitude information generation module provided the preset path, in which the control on sampling frequency, traction speed, turning radius, and positioning accuracy was mainly completed. The position calculation module for the seeding unit in the center of the seeder calculated the position information and heading angle data generated by the latitude and longitude information generation module to obtain the center and radius of the tractor’s movement track and deduced the rotation radius and position of the middle seeding unit. The position calculation module for individual seeding units derived the traction speed, seeding trajectory, seeding rate, and seeding position of individual seeding unit from the rotation radius, position, and heading angle calculated for the middle seeding unit.

**Figure 7:**
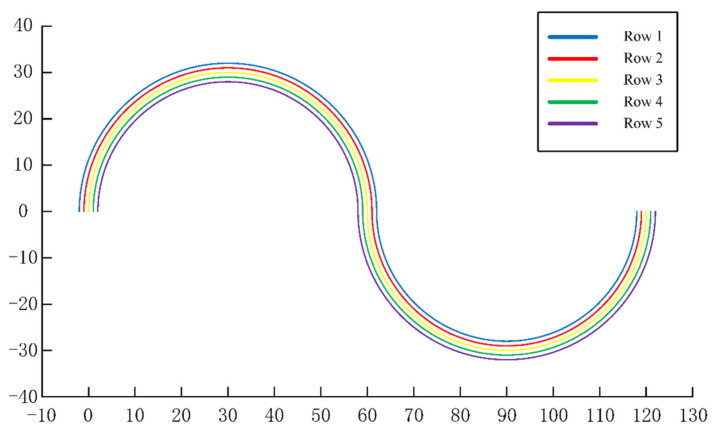
Preset Routes.

The preset route map in this simulation experiment is shown in Figure 7, and the preset values included the distance l between the receiving point and the middle seeding unit, the distance $l_{r}$ between two seeding units, the seeding spacing $l_{0}$, and the turning radius R, as shown in Table 1.

### 3.2. Results and Analysis

This simulation experiment model mainly tested the applicability of this system based on traction speed, number of seeding rows, and positioning accuracy. The number of seeding rows was set to be 5, 7, and 9, with three seeder models. The test of sampling accuracy was set with three accuracy levels, i.e., ±0.01 m, ±0.02 m*,* and ±0.03 m; the test setting of traction speed was set with three speed levels, i.e., 1 m/s, 2 m/s, and 3 m/s. All tests were repeated three times to ensure that the test results were not accidental. In the simulation experiment, the seeding amount of the seeder in the curve area, and the seeding spacing of individual seeding units were recorded, and the seeding qualification rate was calculated (the error between the actual seeding spacing and the specified seeding spacing was less than 0.025 m). The statistical results are shown in Table 2.

Based on the statistical calculation, the theoretical seeding amount in the preset routes corresponding to 5-row, 7-row, and 9-row seeders were 4710, 6594, and 8478, respectively. Taking into account of the analysis data on theoretical seeding amount of in dividual seeding units in Table 2 and Table 3, it was concluded that this model could ensure the seeding amount and uniformity under the set experimental conditions. As shown in Table 4 about the correlation analysis results, there was a significant correlation between positioning accuracy and seeding quality as well as between traction speed and seeding quality.

**Figure 8:**
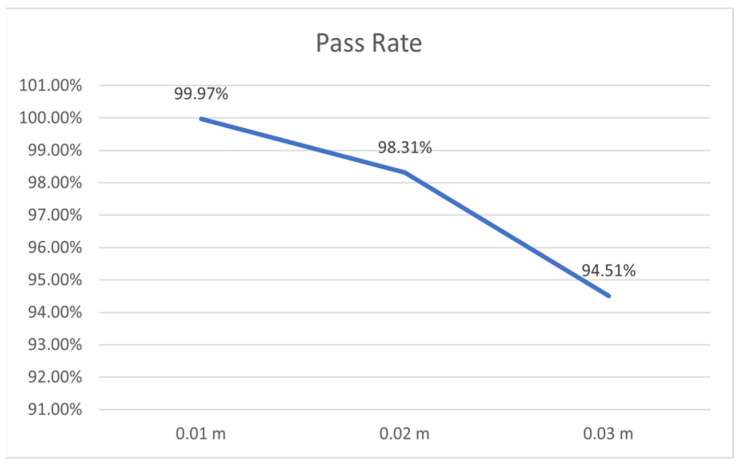
Relationship between Seeding Quality and Positioning Accuracy.

The positioning accuracy showed a significant impact on the seeding quality, and the changing curve is shown in Figure 8. When the number of rows to be seeded is fixed at 5 rows, the seeding qualification rate was increased from 94.51% to 99.97% when the positioning accuracy was increased from ±0.03 m to ±0.01 m, with an increase of 5.46%. Additionally, taking also into account of the analysis result on correlation between positioning accuracy and seeding quality (as high as 0.938) in Table 4, it was concluded that positioning accuracy formed a significant impact on seeding quality in this model. Therefore, it was suggested to use the GNSS receiver with high positioning accuracy (with a positioning accuracy of ±0.01 m) when seeding in the field, which could obtain higher accuracy in the seeding process.

**Figure 9:**
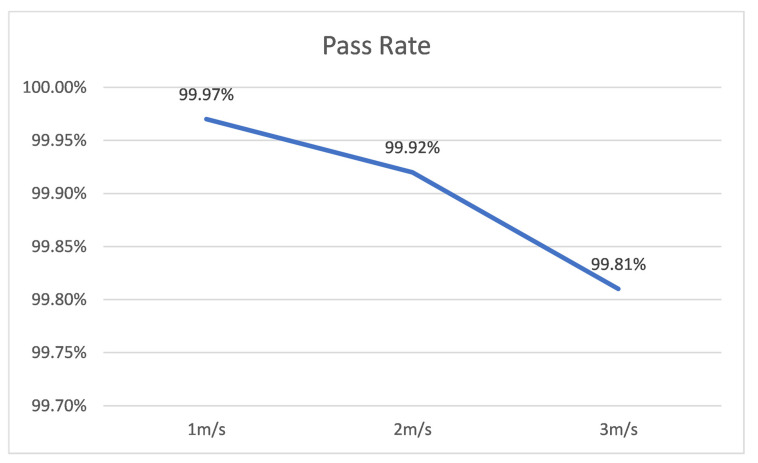
Relationship between Seeding Quality and Traction Speed.

The curve of traction speed versus seeding quality is shown in Figure 9. When the traction speed was decreased from 3 m/s to 1 m/s, the seeding quality accomplished by a 5-row seeder with a positioning accuracy of ±0.01 m increased from the 99.12% to 99.64%. At present, the qualification rate of mechanical corn seeder is 93% when the seeding speed is 5 km/h. With the algorithm in this study, the seeding accuracy rate reached as high as 99.92% when the positioning accuracy was ±0.01 m, and the traction speed was 2 m/s(7.2 km/h), suggesting a significant improvement of the seeding qualification rate. Although the seeding quality showed a downward trend with the increase of speed, it was still higher than the current mainstream mechanical seeding quality of corn. Therefore, when using this model, a lower traction speed should be adopted when possible to balance the seeding quality and the seeding progress.

**Figure 10:**
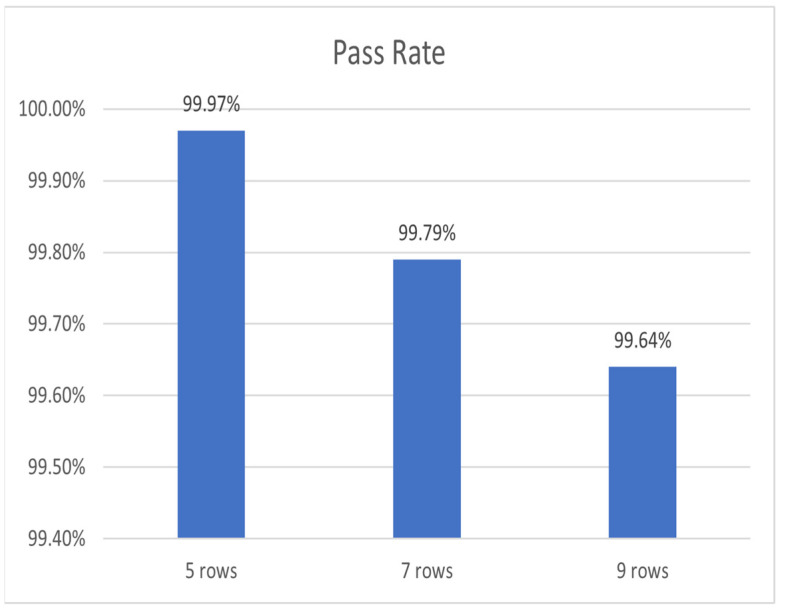
Relationship between Seeding Quality and Number of Seeding Rows.

The influence of the number of seeding rows on seeding quality is shown in Figure 10. When the seeding operation was performed with a positioning accuracy of ±0.01 m and a traction speed of 1 m/s, the qualification rate of the 5-row seeder was 99.97%, and that of the 9-row seeder was 99.64%. Therefore, although the seeding quality decreased slightly with the increase of seeding rows, the correlation between the number of seeding rows and the seeding quality was only 0.73. With the increase of sowing rows, the simulated seeding qualification rate of this model was always higher than 93%, which isthe seeding qualification rate of the mainstream corn seeders in the market. Therefore, this algorithm could be applied to multi-row precision seeders and effectively improve their seeding quality.

This simulation experiment mainly analyzed the influence and correlation of three factors on and with seeding quality, including the number of seeding rows, the traction speed, and the positioning accuracy. The correlation between positioning accuracy and seeding quality was as high as 0.938, suggesting a directly crucial effect of positioning accuracy on the seeding quality. The correlation between traction speed and seeding quality was 0.905, suggesting that the influence of traction speed on seeding quality is also very significant. The correlation between the number of seeding rows and seeding quality was only 0.73, suggesting that the influence of number of seeding rows on seeding quality is not significant. The simulation results showed that when the model was used to seed in the S-shaped curve area with a positioning accuracy of ±0.01 m, a seeding radius of 30 m, and a traction speed of 1 m/s, the seeding qualification rate of individual seeding unit of a 5-row seeder could reach 99.97%, and that of a 9-row seeder could reach 99.64%.When the traction speed was 3 m/s, the seeding qualification rate of individual seeding unit of the 5-row seeder could reach 99.81%, and that of the 9-row seeder could reach 99.12%. Therefore, this algorithm can realize the compensation and positioning functions of a multi-row seeder in its seeding in curve area when the positioning accuracy is ±0.01 m.

## 4. Conclusions

A precise seeding compensation and positioning algorithm based on multi-sensors was proposed in this study, with which the seeding rate and position of individual seeding unit could be calculated in real time according to the specified seeding distance in order to improve the seeding uniformity. According to the simulation results, the proposed algorithm was tested and evaluated. The conclusions are listed as follows:

(1) A multi-sensor precise seeding calculation model was established, with which the real-time speed and position of individual seeding unit could be calculated according to the position and attitude information received by GNSS receiver and angle sensor to predict the next seeding position so as to achieve the goal of uniform seeding in curve area and increase the unit yield of crops.
(2) Simulink simulation software was used to build a simulation model and provide simulation results for analysis in order to determine the correlation of positioning accuracy, traction speed, and number of seeding rows, respectively, with seeding quality. The experimental results showed that the algorithm could achieve the highest seeding qualification rate of 99.81% in the individual seeding units of a 5-row seeder with a positioning accuracy of ±0.01 m and a traction speed of 1 m/s, and it could realize the compensation and positioning functions of a multi-row seeder.

## Figures and Tables

**Table 1:** Parameters of Tractor and Seeder.

Parameters	R	l	$l_{r}$	$l_{0}$
Values	30 m	1.5 m	0.6 m	0.2 m

**Table 2:** Statistical Description of Simulation Experiment.

Positioning	Traction	Seeding	N	Min.	Max.	Average		SD
Accuracy	Speed	Number of Rows	(Statistical Data)	(Statistical Data)	(Statistical Data)	(Statistical Data)	(Standard Error)	(Statistical Data)
0.01 m	1 m/s	5	4686	0.1729	0.237	0.201076	0.000205	0.009923
		7	6560	0.1688	0.2327	0.201061	0.0001754	0.0100477
		9	8450	0.1723	0.2341	0.200732	0.0001525	0.0099144
	2 m/s	5	4706	0.1688	0.2371	0.20023	0.0002246	0.0108954
		7	6580	0.1636	0.236	0.200565	0.0002087	0.0119688
		9	8406	0.1619	0.2402	0.201788	0.0002035	0.0131936
	3 m/s	5	4710	0.1676	0.234	0.200174	0.0002208	0.0107129
		7	6594	0.164	0.2384	0.200172	0.0002135	0.0122606
		9	8470	0.1641	0.2421	0.200361	0.0002113	0.013749
0.02 m	1 m/s	5	4688	0.1556	0.2526	0.201535	0.0003506	0.0169741
		7	6566	0.1535	0.2541	0.201361	0.000304	0.017421
		9	8446	0.1461	0.2518	0.201291	0.0002634	0.0171176
	2 m/s	5	4710	0.1523	0.2491	0.200689	0.0003642	0.0176743
		7	6570	0.1418	0.2638	0.201347	0.0003229	0.0185049
		9	8408	0.1443	0.2581	0.202222	0.0002989	0.0193797
	3 m/s	5	4710	0.148	0.2495	0.200672	0.0003585	0.0173971
		7	6594	0.1412	0.255	0.200686	0.0003195	0.0183435
		9	8470	0.1394	0.2654	0.200823	0.0003041	0.0197878
0.03 m	1 m/s	5	4696	0.1277	0.275	0.20208	0.0005268	0.0255279
		7	6564	0.1218	0.2771	0.202276	0.0004371	0.0250404
		9	8450	0.1304	0.2787	0.202072	0.000382	0.0248306
	2 m/s	5	4708	0.1244	0.2812	0.201541	0.000505	0.0244996
		7	6568	0.1224	0.2842	0.202191	0.0004626	0.0265097
		9	8422	0.1201	0.2875	0.202883	0.0004106	0.0266453
	3 m/s	5	4710	0.1222	0.2749	0.201492	0.0005243	0.0254429
		7	6594	0.1195	0.28	0.201398	0.0004544	0.0260886
		9	8466	0.1257	0.2859	0.201778	0.0004131	0.0268787

**Table 3:** Statistics of Seeding Qualification Rate.

Traction Speed	Positioning Accuracy	Seeding Pass Rate		
		5 Rows	7 Rows	9 Rows
3 m/s	0.01 m	99.81%	99.61%	99.12%
	0.02 m	97.64%	97.02%	96.74%
	0.03 m	93.27%	92.50%	92.07%
2 m/s	0.01 m	99.92%	99.68%	99.51%
	0.02 m	97.95%	97.57%	97.03%
	0.03 m	93.60%	93.85%	92.96%
1 m/s	0.01 m	99.97%	99.79%	99.64%
	0.02 m	98.31%	97.96%	97.88%
	0.03 m	94.51%	94.43%	93.66%

**Table 4:** Analysis on Correlation with Seeding Quality.

Factors	Correlation
Positioning accuracy	0.938
Traction speed	0.905
Number of seeding rows	0.730

## Data Availability

Not applicable.
